# Biosynthesis of Silver Chloride Nanoparticles by Rhizospheric Bacteria and Their Antibacterial Activity against Phytopathogenic Bacterium *Ralstonia solanacearum*

**DOI:** 10.3390/molecules27010224

**Published:** 2021-12-30

**Authors:** Iman Sabah Abd Alamer, Ali Athafah Tomah, Temoor Ahmed, Bin Li, Jingze Zhang

**Affiliations:** 1 State Key Laboratory of Rice Biology, Institute of Biotechnology, Zhejiang University, Hangzhou 310058, China; emansabah29@yahoo.com (I.S.A.A.); alialmalki775@yahoo.com (A.A.T.); temoorahmed@zju.edu.cn (T.A.); libin0571@zju.edu.cn (B.L.); 2 Plant Protection, Agriculture Directorate, AL-Amarah 62001, Iraq; 3 Plant Protection, College of Agriculture, University of Misan, AL-Amarah 62001, Iraq

**Keywords:** *Bacillus mojavensis*, taxonomy, molecular interaction, *Ralstonia solanacearum*, antibacterial activity

## Abstract

*Ralstonia solanacearum* is the most destructive pathogen, causing bacterial wilt disease of eggplant. The present study aimed to develop green synthesis and characterization of silver chloride nanoparticles (AgCl-NPs) by using a native bacterial strain and subsequent evaluation of their antibacterial activity against *R. solanacearum*. Here, a total of 10 bacterial strains were selected for the biosynthesis of AgCl-NPs. Among them, the highest yield occurred in the synthesis of AgCl-NPs using a cell-free aqueous filtrate of strain IMA13. Ultrastructural observation revealed that the AgCl-NPs were spherical and oval with smooth surfaces and 5–35 nm sizes. XRD analysis studies revealed that these particles contained face-centered cubic crystallites of metallic Ag and AgCl. Moreover, FTIR analysis showed the presence of capping proteins, carbohydrates, lipids, and lipopeptide compounds and crystalline structure of AgCl-NPs. On the basis of phylogenetic analysis using a combination of six gene sequences (*16S*, *gyrA*, *rpoB*, *purH*, *polC*, and *groEL*), we identified strain IMA13 as *Bacillus mojavensis*. Three kinds of lipopeptide compounds, namely, bacillomycin D, iturin, and fengycin, forming cell-free supernatant produced by strain IAM13, were identified by MALDI-TOF mass spectrometry. Biogenic AgCl-NPs showed substantial antibacterial activity against *R. solanacearum* at a concentration of 20 µg/mL^−1^. Motility assays showed that the AgCl-NPs significantly inhibited the swarming and swimming motility (61.4 and 55.8%) against *R. solanacearum*. Moreover, SEM and TEM analysis showed that direct interaction of AgCl-NPs with bacterial cells caused rupture of cell wall and cytoplasmic membranes, as well as leakage of nucleic acid materials, which ultimately resulted in the death of *R. solanacearum*. Overall, these findings will help in developing a promising nanopesticide against phytopathogen plant disease management.

## 1. Introduction

*Ralstonia solanacearum* is a soilborne Gram-negative bacterium that causes plant diseases mainly in tropical and subtropical climates [1]. *R. solanacearum* has a wide plant hosts range and it is capable of infecting more than 450 plant species in 54 different botanical families, such as brinjal, tomato, potato, and chili [2,3,4,5]. Bacterial wilt disease caused by *R. solanacearum* is the most infectious soil-borne bacterial disease of eggplant [6]. In addition to the polar flagella responsible for swimming motility, the pathogen produces type IV pili (TFP) that govern twitching motility, which is required for plant colonization and full Virulence [7]. These characters have contributed to the ranking of *R. solanacearum* as one of the most destructive plant-pathogenic bacterial species worldwide [8].

Current resistant cultivars are limited due to the pathogen’s extensive genetic diversity and difficulty in transferring a high number of genes into cultivars with undesirable traits to develop new resistant cultivars [9]. In the last few decades, conventional pesticides in the form of numerous chemicals and antibiotics have been used for the treatment of bacterial wilt disease. However, the long-term use of chemical pesticides leads to resistant strains, environmental pollution, cost increase, and little effectiveness in the field. The biological control methods have been investigated widely for this disease, but many products are just under controlled conditions but not confirmed in the field. In addition, some of the biocontrol products in use were limited by geographical location due to the properties of biocontrol agents. These invited researchers to devise a new strategy to treat the plant diseases that plant pathogens caused, known as the biosynthesis of green nanoparticles [10].

Nanotechnology is the use of matter on an atomic, molecular, and supramolecular scale for industrial purposes, which is considered one of the cutting edge trends successful in many areas, including agriculture. In the last few years, various physicochemical methods have been used to produce metallic NPs; however, they also produce environmentally hazardous residues that are finally released into the environment [11]. To overcome these challenges, biological synthesis that involves the use of living systems (bacteria, fungi, plants, and microalgae) as nanofactories, has emerged as a promising alternative to conventional methods due to their non-toxic, eco-friendly, and more stable nature [12,13]. Among the microorganisms, bacteria occupied the center stage in the nanoparticles synthesis field due to their growing success, ease of handling, and genetic modification [14], and recently, *Bacillus* sp., such as *B. pumilus*, *B. persicus*, and *B. licheniformis*, have received great attention due to their ability in nanoparticles synthesis [15]. The mechanisms of action for nanoparticles against microorganisms is still not entirely clear; however, many researchers have identified some mechanisms, including, most notably, penetration and accumulation of nanoparticles in the cytoplasm, severe damage of cell wall and cytoplasmic membrane [16], generation of reactive oxygen species (ROS) and free radicals, and modulation of microbial signal transduction pathways [17].

The present work aims to screen and identify bacterial strains for efficient synthesis and characterize the AgCl-NPs using scanning electron microscopy (SEM), energy dispersive spectroscopy (EDS), X-ray diffraction (XRD), transmission electron microscope (TEM) and Fourier transform infrared spectroscopy (FTIR), and to assess the in vitro antibacterial activity of biosynthesized AgCl-NPs against phytopathogenic bacterium *R. solanacearum*.

## 2. Results

### 2.1. Bacteria Strains and Biosynthesis of AgCl-NPs

On the basis of characteristics of bacterial colonies such as shape, color, and size, we selected nine strains, named IMA12, IMA13, IMA14, IMA15, IMA16, IMA17, IMA18, IMA19, and IMA20, for the biosynthesis of AgCl-NPs after they were purified.

**Figure 1:**
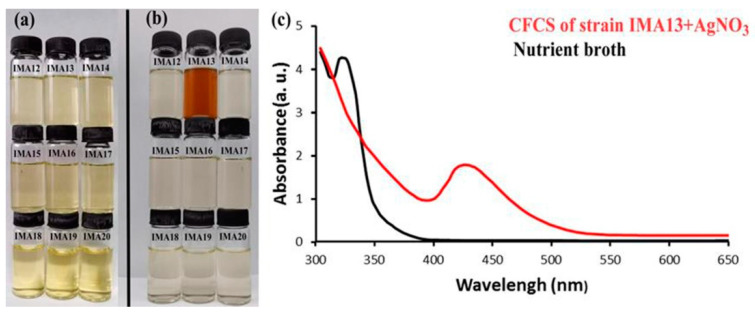
Synthesis of nanoparticles using cell-free culture supernatant (CFCS) of nine bacteria strains and UV-visible spectra of synthesized AgCl-NPs after incubation at 30 °C and 200 rpm for 48 h: (**a**) visual color change before incubation; (**b**) visual color change after incubation for 48 h; (**c**) the absorption spectrum of AgCl-NPs synthesized using IMA13 CFCS, showing a strong peak at 430 nm after 48 h.

The AgCl-NPs formation was observed by visual color change during incubation (Figure 1a,b) and confirmed by UV-visible spectroscopy. The experimental results showed that the obvious color change appeared only in the solution with the CFCS produced by strain IMA13 and AgNO_3_ (Figure 1b), indicating the formation of AgCl-NPs after incubation for 48 h. Subsequently, the repeated assay confirmed that the biosynthesis of AgCl-NPs using the CFCS produced by strain IMA13 was stable and repeatable. No AgCl-NP synthesis was observed in control flasks containing 25 mL of nutrient broth (instead of bacterial supernatant) and AgNO_3_ at 1.0 mM, confirming that extracellular agents of bacterial origin mediated AgCl-NPs synthesis. Analysis of the UV-visible spectra revealed that the sharp surface plasma resonance peaks were at 427 nm after incubation for 48 h (Figure 1c).

### 2.2. Characterizations of AgCl-NPs

The synthesized AgCl-NPs were characterized by scanning electron microscopy (SEM), energy-dispersive spectroscopy (EDS), transmission electron microscopy (TEM), X-ray diffraction (XRD), and Fourier transform infrared (FT-IR) spectroscopy analysis.

**Figure 2:**
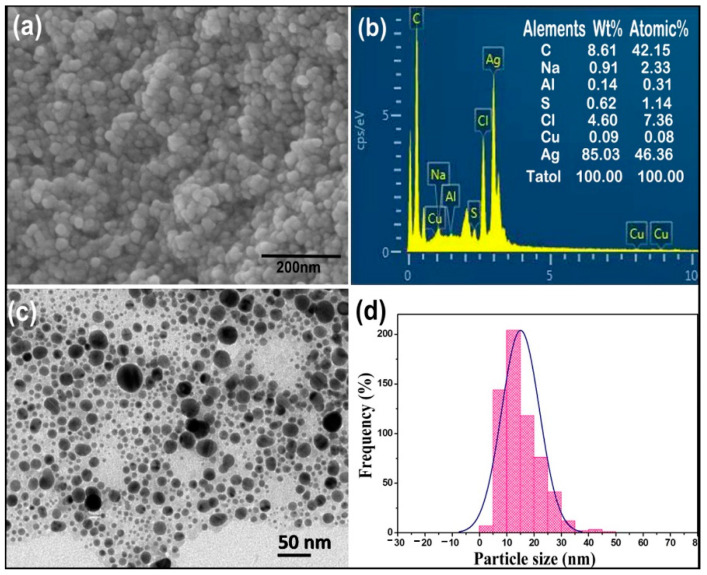
Morphology of silver nanoparticles synthesized using cell-free culture supernatant (CFCS) of strain IMA13: (**a**) SEM; (**b**) EDS; (**c**) TEM; (**d**) distribution frequency (%) of AgCl-NPs particle size.

The SEM micrographs showed that the external surfaces of spherical AgCl-NPs synthesized by cell-free culture supernatant of strain IMA13 were smooth (Figure 2a). The EDS analysis of AgCl-NPs revealed the pure silver (46.36%) was the uppermost major constituent element at 3 keV, while carbon was the second major element at 0.5 keV compared to Na, Cl, S, Al, and Cu between 0 and 9 keV (Figure 2b), confirming the existence of the silver element in the synthesized AgCl-NPs (including AgClNPs). The TEM micrograph showed that the different sizes and shapes of AgCl-NPs ranged from 5 to 35 nm (Figure 2c,d). The micrograph also showed that the majority of AgCl-NPs were spherical, while others were oval-shaped (Figure 2c).

**Figure 3:**
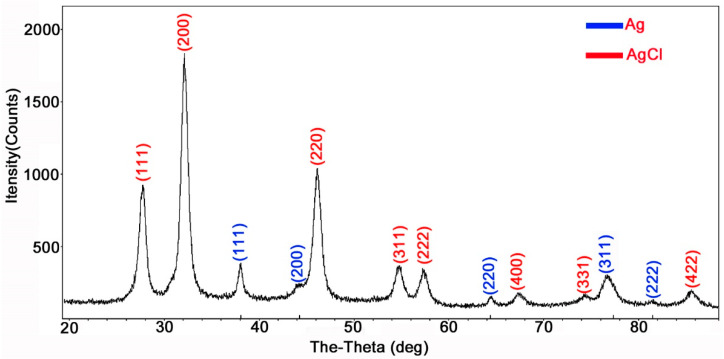
X-ray diffraction patterns of the biosynthesized AgCl-NPs by CFCS extract of bacterium strain IMA13.

The XRD analysis of AgCl-NPs produced had recorded the diffraction five peaks in the 2*θ* range of a rounded 38.17°, 44.37°, 64.55°, 77.54°, and 81.69° corresponded to the silver crystal planes (111), (200), (220), (311) and (222) (Figure 3), respectively. All these diffraction peaks are well matched to the face-centered cubic (FCC) phase with the quality value to the Silver by Inorganic Crystal Structure Database (ISCD) with the silver file (no. 64994). Moreover, the XRD analysis also showed nine peaks at 27.84°, 32.25°, 46.26°, 54.86°, 57.52°, 67.49°, 74.51°, and 85.75° (Figure 3). These peaks showed a proper match with the reference peak positions of the face-centered cubic (FCC) structure of AgCl through comparing it with (ISCD no. 56538). Because AgCl-NPs account for 95.7% compared with Ag-NPs 5.3% in XRD analysis, the content became the synthesis of AgCl-NPs.

**Figure 4:**
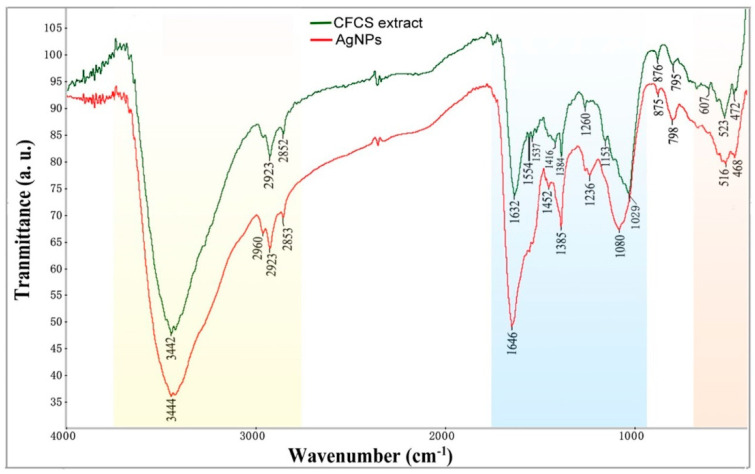
Characterization of the biosynthesized AgCl-NPs using cell-free culture supernatant extract of bacterial strain IMA13. FTIR of biosynthesized AgCl-NPs (red color), and cell-free culture supernatant of bacterium strain IMA13 dry (black color).

The FTIR spectra were recorded for identifying the functional groups involved in the AgCl-NP reduction (Figure 4). The broad and strong bands at 3444 cm^−1^ were due to the bonded amine groups (–NH) in the interaction of bacterial extract with AgCl-NPs powder. The peaks that appeared at 2923 cm^−1^ were attributed to symmetric and asymmetric CH_2_, stretching modes of carbohydrates and fatty acids. The stronger peaks at 1646 cm^−1^ were attributed to the C=O (carboxylic group) and amide I group stretching vibrations. The peak at 1385 cm^−1^ was assigned to the methyl rocking vibrations (CH_3_ of hydrocarbons). The peak near 1080 cm^−1^ was attributed to P=O stretching vibrations in phospholipids and C=O stretching vibrations in polysaccharides (glycosidic bonds and pyranoid rings) [18]. The peak at 516 cm^−1^ in the low wavenumber was considered to be the presence of Van der Waals forces of interaction between oxygen groups in protein structures and in the CFCS extract on the surface of AgCl-NPs [19].

On the basis of characteristics of FTIR spectra (Figure 4), we analyzed the shift changes between AgCl-NPs powder and the CFCS extract of bacterial strain IMA13. The peak at 3444 cm^−1^ with a shift change (−2 cm^−1^, compared to bacterial extract) revealed that the AgCl-NPs interacted with the proteins of the CFCS extract. The peak at 1646 cm^−1^ with a large shift change (−14 cm^−1^) showed that the AgCl-NPs strongly bonded with negatively charged carboxylic or amide I groups in proteins. The stronger peak at 1080 cm^−1^ with a largest shift change (−51 cm^−1^) revealed that the AgCl-NPs strongly interacted with lipids and carbohydrates. In addition, the peak at 516 cm^−1^ with a shift change (−7 cm^−1^) could also display the electrostatics interactions of nanoparticles with oxygen from hydroxyl groups. These biological components may play a significant role in forming and stabilizing nanoparticles.

### 2.3. Identification of Strain

**Figure 5:**
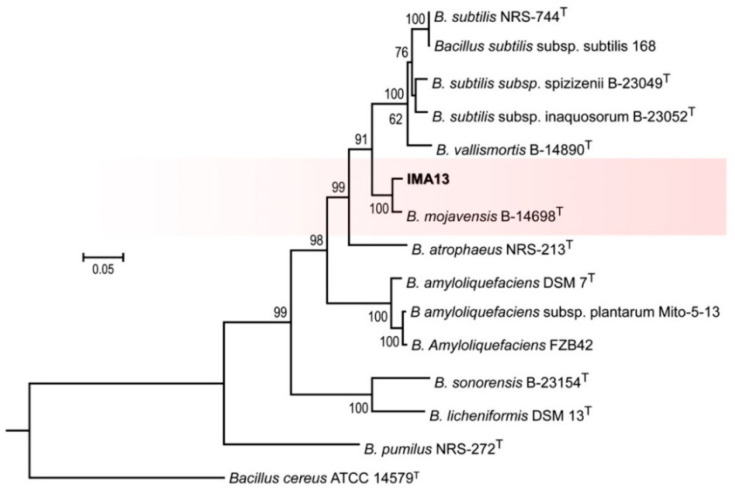
Maximum likelihood (ML) tree generated from a combination of gyrA, rpoB, purH, polC, and groEL gene sequences of 15 taxa. The tree is rooted with *B. cereus*. The type strains are indicated with T. Strains in this study are showed in bold.

The strain IMA13 was firstly identified by sequence analysis of *16S* rRNA gene. The result of a BLAST search showed that the strain IMA13 had identities of 100% for the *Bacillus* sp. (GenBank no. MG470688.1) and *Bacillus halotolerans* (GenBank no. MN330417.1). To delineate species boundaries, we amplified five genes (*gyrA*, *rpoB*, *purH*, *polC*, and *groEL*) (Table 1); all six gene sequences were deposited in the GenBank database (Table 2), and a phylogenetic tree was constructed by using combination of six gene sequences (*16S*, *gyrA*, *rpoB*, *purH*, *polC*, and *groEL*) and the *B. cereus* ATCC 14579T was used as the outgroup. The phylogenetic tree showed that all studied isolates were separated into different clades (Figure 5). The relationship of almost all reference isolates could be clearly distinguished on the level of species. The strain IMA13 clustered with *B. mojavensis* B-14698T as a clade with 100% bootstrap support. In addition, the topology of ML tree analysis was congruent with the results that reported by [20]. Therefore, strain IMA13 was identified as *B. mojavensis*.

### 2.4. Analysis of Lipopeptide Compound

**Figure 6:**
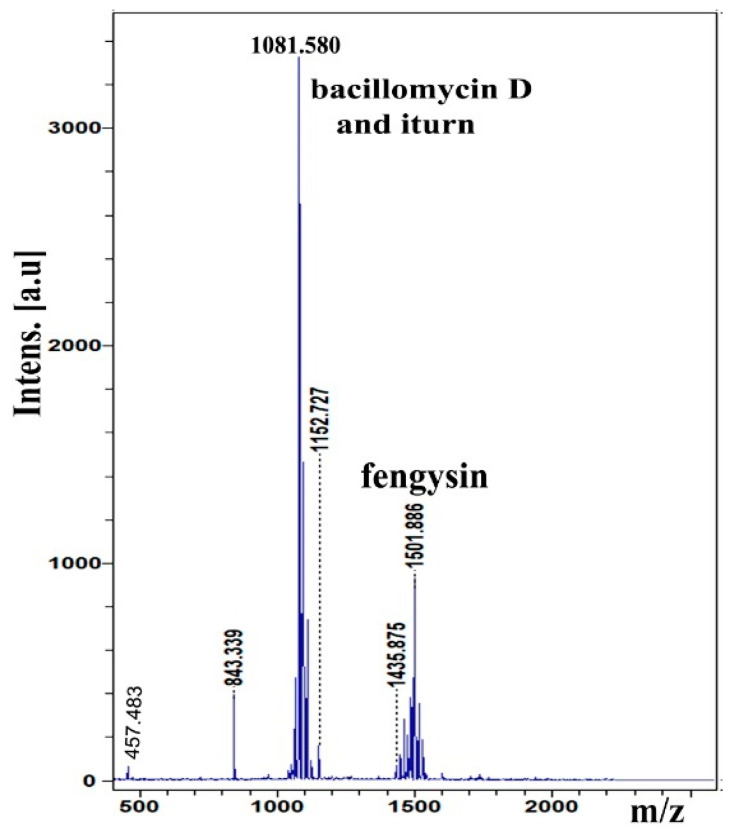
MALDI-TOF MS analysis of lipopeptide compounds produced by IMA13 strain.

The MALDI-TOF MS was applied to detect and identify lipopeptide compounds from whole cells of the IAM13 strain. The MALDI-TOF analysis results showed the major peaks revealed the presence of bacillomycin D, iturin, and fengycin (Figure 6). The peaks at *m*/*z* 1053.5, 1067.5, 1081.5, and 1095.6 were contributed to by bacillomycin D corresponding to C_14–17_ bacillomycin D [M + Na]^+^, respectively (Table S1) [21]. The peaks at *m*/*z* 1065.5, 1079.6, 1095.6, 1109.6, 1123.6, and 1152.7 corresponded to C_14–15_ iturin [M + Na]^+^, C_15–17_ iturin [M + K]^+^, and C20 iturin [M + Na]^+^, respectively [22,23]. Moreover, the peaks at *m*/*z* 1435.9, 1449.9, 1463.9, 1473.8, 1477.9, 1485.9, 1487.8, 1501.8, and 1515.9 corresponded to C14–16 fengycin [M + H]^+^, C14 fengycin [M + K]^+^, C16 fengycin [M + Na]^+^, and C16–17 fengycin [M + K]^+^, respectively [24,25]. 

### 2.5. Antibacterial Activity of AgCl-NPs

**Figure 7:**
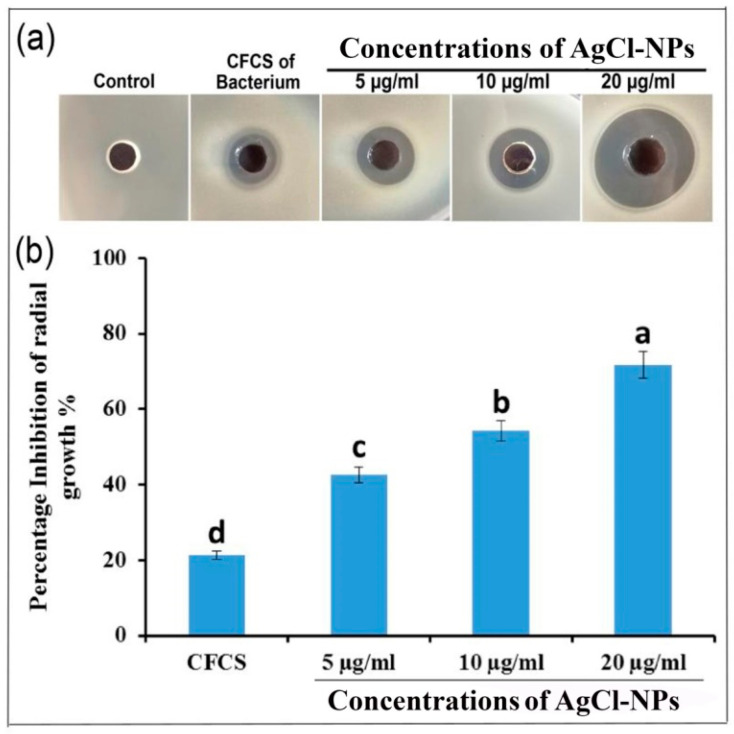
Antibacterial activity of the biosynthesized AgCl-NPs against phytopathogenic bacterium strain YY06: (**a**) inhibitory activity of different concentrations of AgCl-NPs against bacterial growth on LB agar; (**b**) percentage of bacterial growth inhibition of different concentrations of AgCl-NPs. Vertical bars represent standard errors of the means (*n* = 3). The bars with different letters are significantly different (*p* < 0.05). CFCS: cell-free culture supernatant.

The antibacterial activity of AgCl-NPs synthesized using a CFCS extract of bacterial *B. mojavensis* IMA13 against phytopathogenic bacterium *R. solanacearum* YY06 was investigated by agar well diffusion assay. The result showed the biggest percentage inhibition of radial growth was 75.8% at concentration of 20 μg/mL^−1^, followed by 47.6% and 37.1% at the concentrations of 10 and 5 μg/mL^−1^, respectively (Figure 7a). The statistical analysis confirmed that the antibacterial activity of AgCl-NPs among different treatments was strikingly different (*p* < 0.05) (Figure 7b). In addition, CFCS extract of bacterial strain IMA13 also had markedly inhibitory activity compared to control.

**Figure 8:**
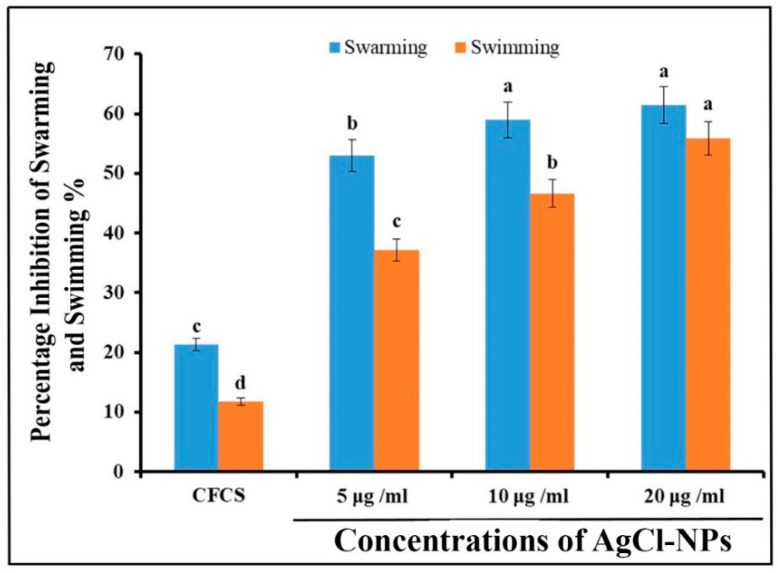
Effect of biosynthesized AgCl-NPs on the motility of *R. solanacearum* after incubation for 96 h. Vertical bars represent swimming (red color) and swarming (blue color). Vertical bars represent standard errors of the means (*n* = 3). The bars with different letters are significantly different (*p* < 0.05). CFCS: cell-free culture supernatant.

To determine effects of synthesized AgCl-NPs on *R. solanacearum* motility, we performed the swarming and swimming motility assay in the media with agar of different concentrations. As shown in Figure 8, the results showed the percentage inhibition of swarming motility were 61.43, 58.93, and 52.98% at concentrations of 20, 10, and 5 μg/mL^−1^ AgCl-NPs, respectively, after 72 h. Although the highest inhibitory activity was at a concentration of 20 μg/mL^−1^ AgCl-NPs, there was no significant difference between 10 and 20 μg/mL^−1^ AgCl-NPs (*p* < 0.05). Moreover, the results were showed the ability of AgCl-NPs on inhibition of the swimming motility of *R. solanacearum*, where the percentage inhibition of swimming motility reached to 55.8, 46.6, and 37.1% at concentrations of 20, 10, and 5 μg/mL^−1^ AgCl-NPs, respectively. In addition, The CFCS extract of strain IMA13 also displayed obviously inhibitory activity against *R. solanacearum* swarming and swimming motility compared to control (ddH_2_O) (Figure 8).

**Figure 9:**
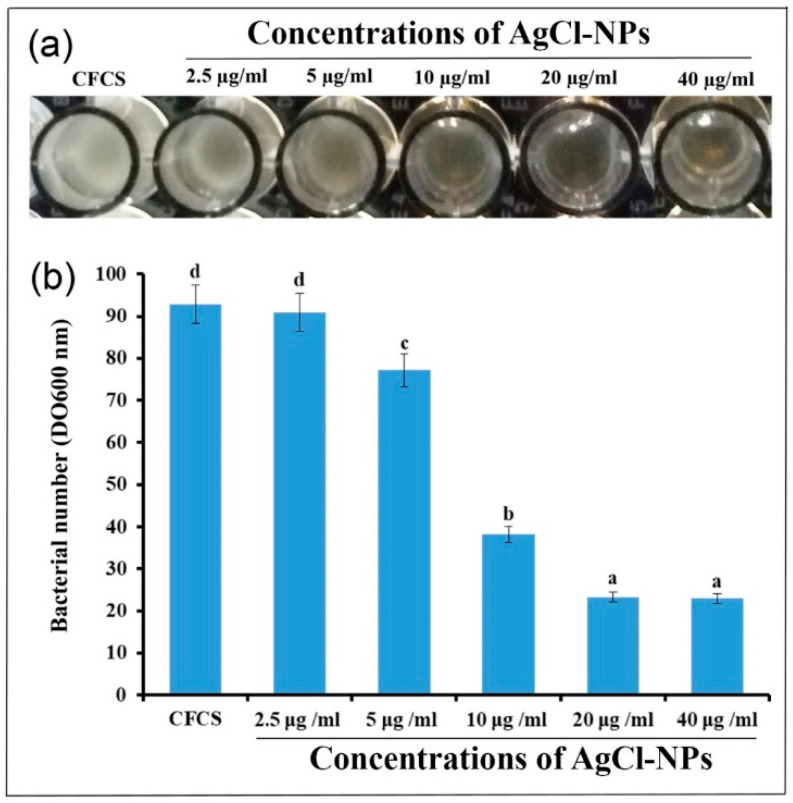
Determination of minimum inhibition concentration of biosynthesized AgCl-NPs by CFCS of bacterium strain IMA13 against bacterium phytopathogenic strain YY06: (**a**) the visible bacterial concentration decreased from left to right on one row of the 96-well microtiter plate; (**b**) inhibitory activity of different concentrations of AgCl-NPs. Vertical bars represent standard errors of the means (*n* = 3). The different letters are significantly different (*p* < 0.05) by LSD test; values are average of three replicates.

In vitro MIC results indicated that the AgCl-NPs significantly inhibited the growth of phytopathogenic strain YY06 after incubation of 48 h (Figure 9). The five varying concentrations of AgCl-NP suspension of 2.5, 5, 10, 20, and 40 µg/mL^−1^ caused a reduction in the OD_600_ values of bacterium strain YY06 to 23.32, 38.16, 77.15, 90.95, and 92.87%, respectively, compared to the CFCS, which was 22.99% (Figure 9a,b). The AgCl-NPs exhibited the lowest MIC against strain YY06 5 μg/mL^−1^, suggesting good antibacterial potential against the bacterium studied.

### 2.6. Ultrastructural Characteristics of AgCl-NPs Interaction with Pathogen

**Figure 10:**
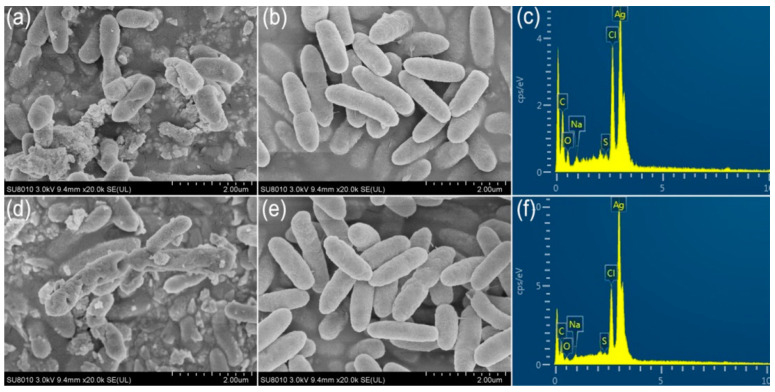
The micrographs of scanning electron microscopy and energy-dispersive spectroscopy (**a**,**b**,**d**,**e**): (**a**,**b**) SEM analysis; (**a**) treatment by AgCl-NPs and (**b**) CFCS extract for 4 h; (**c**) EDS analysis in (**a**); (**d**,**e**) SEM analysis; (**d**) treatment by AgCl-NPs and (**e**) CFCS extract for 8 h; (**f**) EDS analysis in (**d**).

To observe the interaction of the AgCl-NPs with bacteria cells, we observed morphological changes on the surfaces of the cells of *R. solanacearum* YY06 by SEM after cells were treated at a concentration of the 20 µg/mL^−1^ of AgCl-NPs for 4 and 8 h. The micrographs of SEM showed that the small lamellar fragments appeared on the surfaces of some cells, whereas the sunken sites around micropores or fissures on the surfaces were also observed after treatment for 4 h (Figure 10a). After treatment for 8 h, deeply rounded holes with different sizes formed on the surfaces of some cells, resulting in the cell deformation (Figure 10d), compared to control (Figure 10b,e). The energy-dispersive spectroscopy (EDS) analysis indicated the presence and accumulation of Ag as well as O, N, C, and S elements on the surfaces of bacterial cells after on samples treated for 4 or 8 h (Figure 10c,f).

**Figure 11:**
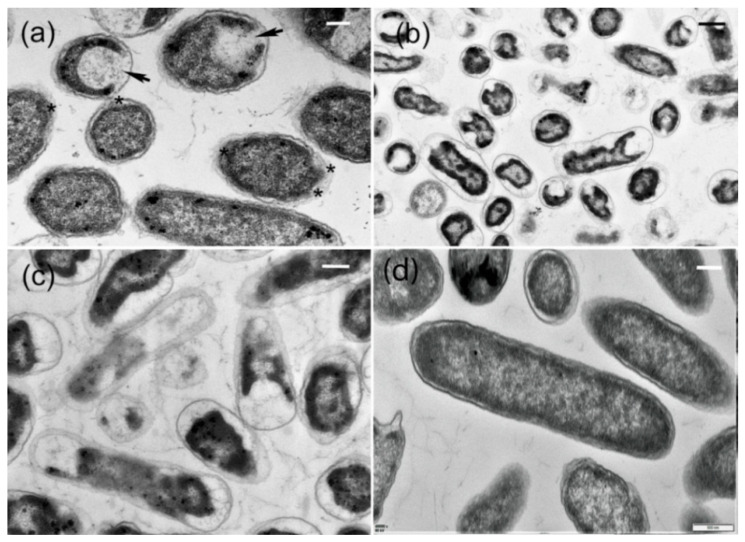
Ultrastructural characteristics of interaction of Ag-NPs with cells of *R. solanacearum*; (**a**) interruption (*) of the local cell walls and partial degradation (arrows) of cytoplasmic membranes in some cells after treatment by AgCl-NPs (**b**) for 4 h; (**b**,**c**) treatment by AgCl-NPs for 8 h; (**b**) change of structural components of the cytoplasm in some cells; (**c**) change of cell walls and the cytoplasm in some cells; (**d**) treatment by CFCS extract (control) for 8 h.

The TEM micrographs showed that the response of *R. solanacearum* to AgCl-NPs varied in different cells, including damage of cell walls and cytoplasmic membrane, as well as cell deformation. After treatment by AgCl-NPs for 4 h, interruption of the local cell walls occurred in most bacterial cells, which looked perforated, or the other cell walls became wavy (Figure 11a). During this period, partial degradation of cytoplasmic membranes was surrounded by a few cells (Figure 11a). After 8 h, enhancement of morphological changes was observed. The lesser cell shapes were deformed with the decreased sizes, and most remained unchanged (Figure 11b). However, the structural components of the cytoplasm aggregated in clumps of high electron density located in electron-lucent cytoplasm. These changes could be related to the disintegration of bacterial cells. Figure 11c demonstrated that the electron-dense cell walls and cytoplasm became very electron-lucent in some cells. During this period, widening of some cell walls with blurred outlines was especially marked compared to control in Figure 11d.

## 3. Discussion

Recently, a new approach using microbes, such as bacteria, has attracted the attention of the scientific community for NP fabrication owing to their eco-friendly, non-toxic, and stable nature as compared with previously available expensive and environmentally corrosive physico-chemical methods. Biologically synthesized AgCl-NPs are promising application prospects in the control of pathogenic microorganisms in the areas of health and agriculture [26]. Although several nanoparticles have been biosynthesized using bacteria for their ability to inhibit plant pathogens, the investigation for new nanoparticles with specific physicochemical and biological properties remains at the forefront of nanotechnological research [15]. The biological components of strain IMA13 extract, such as proteins, lipids, carbohydrates, and lipopeptide compounds, were involved in interaction with AgCl-NPs for stabilization of nanoparticles. The formation of these AgCl particles could be due to the interaction of the NaCl content of nutrient broth with silver ions, which resulted in formation of AgCl-NPs. However, synthetic yield is involved in components of secondary metabolites secreted by microorganisms and synthesis mechanisms of AgCl-NPs [27]. In this study, among the nine bacterial strains, only *Bacillus mojavensis* IMA13 from rhizospheric soil of eggplants indicated the formation of Ag/AgClNPs after incubation for 48 h (Figure 1b). AgCl-NPs were produced due to presence of NaCl in medium. Synthesis of AgCl-NPs from a silver nitrate precursor as mediated by supernatant of several microbes has been reported as the fungi such as *Macrophomina phaseolina* [28], yeasts such as *Meyerozyma guilliermondii* KX008616 [29], and bacteria such as *Raoultella planticola* and *Pantoea agglomerans* [30]. However, the silver chloride nanoparticles synthesis by supernatant of bacteria *B. mojavensis* was first reported.

Few studies show biogenic AgCl-NPs have antibacterial activity [31]. Therefore, in this study, we provided an example of the activity of Ag/AgClNP against bacterial pathogen. Moreover, biosynthesized AgCl-NPs were characterized with standard material characterization techniques, viz., XRD, FTIR, SEM, TEM, and EDS. In the present study, the crystallographic planes peaks in the XRD pattern (Figure 3) showed that the prepared crystalline structure of biogenic AgCl-NPs is a mixture of metallic Ag and AgCl [32,33]. Previous research had shown that *B. mojavensis* was for the synthesis of AgCl-NPs [34,35], but the size and shape of synthesized AgNPs depended on the different strains. The *B. mojavensis* BTCB15 produced AgNPs of 105 nm size [35], and the 32A produced AgNPs ranging in size from 6 to 72 nm with hexagonal and cubic crystal configurations [34]. In this study, the strain IMA13 produced the spherical and oval AgNPs with 5–35 nm sizes, being different in previous reports. However, the difference in size and shape of AgNPs synthesized using different strains of the same species could be related to that of metabolites among different strains.

In *Bacillus*, lipopeptides are the most known due to their antimicrobial and antiviral properties. On the basis of analysis of *B. mojavensis* RRC101 genome, it was found to possess biocontrol-relevant secondary metabolism pathways, including fengycins, surfactins, subtilosin, bacilysin, bacillomycin D, and bacillibactin [36]. This characterization of lipopeptides has been confirmed [37,38,39]. The researcher found that the strain *B. mojavensis* B0621A produced a new iturinic lipopeptide called mojavensin A, but species identification could need to be verified further [39]. In this study, cell-free culture supernatant (CFCS) secreted by *B. mojavensis* IMA13 only found three types of lipopeptide compounds: bacillomycins D, iturins, and fengycins. Although the peak of the FTIR spectra at 1646 cm^−1^ showed that the AgCl-NPs strongly bonded with a negatively charged carboxylic group or amide I group in proteins, lipopeptide compounds possibly took part in the synthesis of AgCl-NPs due to the presence of the carboxylic group and carbonyl group. Lipopeptide compounds possibly took part in the formation of the AgCl-NPs due to amino acid residues with C=O groups in fengycins (Glu), bacillomycin D (Asn), and iturins (Asn and Glu), as well as the larger shift change on peak at 1646 cm^−1^. Similarly, the largest shift in the peak at 1029 cm^−1^ towards lower frequency compared to peak in 1080 cm^−1^ was attributed to the binding of C–C–O and C–C–H groups with nanoparticles [19]. This was very likely, except with lipids and carbohydrates, lipopeptide compounds were involved in formation of the AgCl-NPs due to the AgCl-NPs binding with hydroxyl group on amino acid residues from fengycins (Thr and Tyr), bacillomycin D (Ser, Thr, and Tyr), and ba Iturins (Ser and Tyr). However, it is well known that the proteins bind with nanoparticles through cysteine residues or free amino groups [40]. Electrostatic interactions of negatively charged carboxyl groups in proteins or in heterocyclic compounds also bind with nanoparticles [41].

In addition, some studies show the antibacterial effect of AgCl-NPs depended strongly on the size and the shape of the particles [42], while spherical AgCl-NPs were more stable and had a higher antibacterial activity compared to other shapes [43,44]. Thus, presumably, the AgCl-NPs synthesized by strain IMA13 have higher antibacterial activity. However, due to differences of different test methods, antibacterial effectiveness against different pathogens among strains still cannot be well evaluated only on the basis of data from the literature; however, this study provides important information for biosynthesis of the AgCl-NPs.

The AgNPs exhibit their antimicrobial potential through multifaceted mechanisms due to their antimicrobial activity against a diverse and broad range of plant pathogens [45]. In this study, the biosynthesized AgNPs exhibited high inhibitory activity against growth, swarming, and swimming motility of *R. solanacearum* and indicted its potential applications against bacterial wilt disease, which is similar to a previous report [46]. Meanwhile, the mode of AgNPs action on bacterial cells was characterized by SEM on the basis of the morphological changes of cells, and the presence of nanoparticles was confirmed by EDS, providing evidence of direct physical interaction between nanoparticles and bacterial cells (Figure 10a,d). Similarly, TEM micrographs revealed that the AgNPs caused damage to cell walls and the cytoplasmic membrane, as well as cell deformation, finally leading to the disintegration of bacterial cells (Figure 11a–d). Therefore, this study extends our understanding of nanoparticles that could potentially be used as an effective strategy for preventing diversified bacterial diseases.

## 4. Materials and Methods

### 4.1. Sample Collection and Bacterial Isolation

The soil samples were collected from rhizospheric soil of eggplants in different fields in Hangzhou city, Zhejiang Province, China, on 20 June 2017 by Abd Alamer et al. [45], wherein eggplant plants had been grown in fields that were heavily infected by *R. solanacearum* for many years. The annual average temperature in this area is 15.9–17 °C, and average humidity is 76–81%. The bacterial strains isolated previously were stored in 20% glycerol. For this study, they were cultured and purified using the streaking plate method on Luria Broth (LB) agar plates at 28 ± 2 °C, as described by Abd Alamer et al. [45]. 

### 4.2. Biosynthesis of AgCl-NPs

For the cell-free culture supernatant (CFCS) of each strain, nine pure bacterial strains were cultured in nutrient broth (10 g tryptone, 3 g beef extract, 2.5 g glucose, and 5 g NaCl per liter; pH 7.2) in a ZWY-211B rotary shaker at 30 °C and 200 rpm for 24 h, and the CFCS was obtained by centrifugation and filtration with a millipore membrane with 0.22 µm pore size. For biosynthesis of AgCl-NPs, 35 mL of the CFCS was mixed with 65 mL (1.0 mM) of AgNO_3_ solution (Sinopharm Chemical Reagent Co., Ltd., Shanghai, China) prepared with double-distilled water (ddH_2_O) in a 250 mL Erlenmeyer flask, as described by [15]. The flasks were kept in the dark rotary shaker at 30 °C and 200 rpm for 48 h, and color change in each flask was observed. If the flasks had a strong color change (for example, from yellowish to red-brown color), the corresponding strains were used to re-synthesize the AgCl-NPs. To confirm the formation of AgCl-NPs, we measured synthesized solution using a UV2550 UV–VIS spectrophotometer (Shimadzu, Kyoto, Japan) from 200 to 800 nm at 1 nm resolution. The experimental flasks without the silver ion but with bacterial metabolites were used for control, and the experiments were conducted in triplicate. The brown-reddish solution was centrifuged at 20,000 rpm for 15 min, and precipitate was washed twice with ddH_2_O, and they were freeze-dried by Alpha 1–2 LDplus dryer (Osterode, Germany) into AgCl-NPs powder for characterization study.

### 4.3. Characterizations of AgCl-NPs

A characterization of the synthesized AgCl-NPs was examined, as described by [10]. Surface characteristics and size of AgCl-NPs were observed using scanning electron microscopy (SEM) (Zeiss Gemini SEM 300, Jena, Germany). The nano-silver element density was confirmed using an energy dispersive spectroscopy (EDS) detector (Oxford Instruments, Oxford, United Kingdom) at 20 keV. The shape and size of AgCl-NPs were detected by transmission electron microscopy (TEM) (JEM-1200EX, Tokyo, Japan). In addition, the crystalline nature of AgCl-NPs was detected by X-ray diffraction (XRD) analysis using a Siemens D5000 diffractometer in the 2θ range from 10° to 90° with Cu-K radiation at an operating voltage of 45 kV and a current of 0.8 mA (Munich, Germany). The dried CFCS extract with or without AgCl-NPs was analyzed using a Fourier transform infrared (FTIR) spectrometer (Bruker Vector 22, Ettlingen, Germany) in the mid-infrared light region of 490–4000 cm^−1^ with a resolution of 4 cm^−1^.

### 4.4. Identification of Bacterium Strain IMA13

To identify the bacteria strain with effectively synthesized AgCl-NPs, we synthesized the six primer pairs in Table 1. The bacterial strain was cultured on LB at 30 °C for 24 h, and the chosen primers were amplified in an automated thermal cycler (Eppendorf AG, Hamburg, Germany). The *16S* rRNA gene amplification was carried out according to the method described by [47]. The sequences of gyrase subunit A (*gyrA*), RNA polymerase subunit B (*rpoB*), phosphoribosylaminoimidazolecarboxamide formyltransferase (*purH*), DNA polymerase III subunit alpha (*polC*), and 60 kDa heat-shock protein (*groEL*) genes were amplified, as described by [20]. The amplified products were checked on a 1% agarose gel under an ultraviolet transilluminator (GenoSens 1850, Clinx Science Instruments Co., Ltd., Shanghai, China), and the eligible products were submitted to the Sangon Biotech Company Limited (Shanghai, China) using the Sanger method for sequencing of both strands on automated DNA sequencer (ABI 3730xl, Applied Biosystems, Foster City, CA, USA).

### 4.5. Phylogenetic Analysis

For phylogenetic analysis, sequences of six genes (*16S*, *gyrA*, *rpoB*, *purH*, *polC*, and *groEL*) were assembled and edited using BioEdit software [48], and they were deposited in the GenBank sequence database. The closely related genes downloaded and *Bacillus cereus* strain ATCC 14579 was used as the outgroup (Table 2).

Sequences of each gene were aligned with MAFFT 7.273 [49], and the resulted alignment was put into Gblocks 0.91b to eliminate the ambiguously aligned positions and divergent regions before phylogenetic analyses [50]. Molecular phylogenies with maximum likelihood (ML) were constructed by RaxmlGUI v. 1.5 [51]. ML bootstrap analysis for each ML tree was performed with 1000 fast bootstrap replicates with the same parameter settings using the GTR+I+G model of nucleotide substitution. A threshold of ≥50% was used as the cut-off for significantly supported nodes.

### 4.6. Lipopeptide Identification 

To identify the lipopeptides compounds produced by strain IAM13, we used the method described previously [45]. Briefly, the strain IMA13 was grown on an LB agar plate at 30 °C for 24 h. Then, the colony was transferred to an Eppendorf tube containing a matrix solution composed of cyano-4-hydroxycinnamic acid in 70% water, 30% acetonitrile, and 0.1% trifluoroacetic acid (*v*/*v*). Bacterial cells were shaken for their homogeneity and then centrifuged at 5000 rpm. A total of 1 μL of the cell-free supernatant was put onto the targeted plate of MALDI-TOF MTP 384 (Bruker ultra flextreme instrument) and allowed to dry. The spectra analysis was conducted using an ultra-extreme instrument MALDI-TOF (Bruker, Bremen, Germany) with a Scout-mtp ion source equipped with a 337 nm nitrogen laser. All spectra were acquired in the reflector positive ion mode and the acceleration and reflector voltages were 25 kV and matrix suppression in deflex-ion mode at *m*/*z* 750. The laser power was set to just above the ionization threshold (around 35%). The resulting sample spectra from 1000 laser shots per *m*/*z* segment were displayed in 10 groups of 50 shots distributed in three different locations on the surface of a matrix spot. Spectra were detected in the positive and reflector mode in 500 to 5000 Da.

### 4.7. Antibacterial Activity of AgCl-NPs

#### 4.7.1. Agar Well Diffusion Assay

To determine antibacterial activity of synthesized AgCl-NPs, we used agar well diffusion technique, as described by [52], to test activity against bacterial pathogen *Ralstonia solanacearum* YY06 (a highly aggressive) from the Plant Pathology Department, Zhejiang University, China. Briefly, 1.0 mL of strain YY06 (about 1 × 10^8^ CFU/mL) grown in LB broth at 200 rpm and 30 °C for 24 h was mixed with 15 mL LB agar at 45 °C in a 9 cm Petri dishes. After solidification under fume hood, a well was prepared using a sterile cork borer of 5 mm diameter on the center of a Petri dish, and 30 µL of AgCl-NPs solution at concentrations of 5, 10, and 20 µg/mL^−1^ was added into wells. The 30 µL of the CFCS or ddH_2_O was used as controls. Antibacterial activity was determined by diameter measurement of inhibition zone around the each well and then calculated as percentage inhibition of radial growth (PIRG %) by using the formula following (PIRG (%) = [(treatment − control (CFCS))/control] × 100%). Each treatment was replicated three times.

#### 4.7.2. Motility Assay

The effect of synthesized AgCl-NPs solution on the swarming motility of cells of strain YY06 was examined in the LB broth supplemented with 0.7% (*w*/*v*) agar [53], and on swimming motility of the test bacteria in LB broth containing 0.3% agar [27]. Briefly, 5, 10, and 20 µg/mL^−1^ of synthesized AgCl-NP solution was mixed with both all LB media at 45 °C in separate petri plates, and 30 µL of the CFCS or ddH_2_O was used as controls. After solidification of media under fume hood, 5 µL of cell suspension of strain YY06 (about 1 × 10^8^ CFU/mL) grown in LB broth at 200 rpm and 30 °C for 24 h was dropped to the center of each plate and then incubated for 3 days at 30 °C. The swarming and swimming motility of cells of strain YY06 was determined by measuring the colony diameters [54]. Percentage inhibition was calculated by using the formula, as described previously [55]. Each treatment was replicated three times.

#### 4.7.3. Determination of Minimum Inhibitory Concentration (MIC)

To determine the lowest concentration of the AgCl-NPs synthesized by the CFCS extract of strain IMA13 for inhibiting the visible growth of plant pathogenic bacterium strain YY06, we carried out the MIC assay using the 96-well microtiter plate method, as described by [56]. Briefly, 200 μL of the synthesized AgCl-NPs (in LB broth) were added in 96-well plates with decreasing concentrations (2.5, 5, 10, 20, and 40 µg/mL^−1^). A total of 200 μL of LB broth with 30 µL of ddH_2_O or with 30 µL of CFCS extract of strain IMA13 was used as controls. Each well of the microtiter plates were inoculated with 10 μL suspension (about 1 × 10^8^ CFU/mL) of strain YY06 grown in LB broth at 200 rpm and 30 °C for 24 h, and then the microtiter plates were incubated in a rotary shaker at 30 °C and 200 rpm for 48 h. The optical density of bacterial cells was measured using a Spectra Max spectrophotometer (Molecular Devices, Sunnyvale, CA, USA) at 600 nm (OD_600_). Three replicates for each treatment were used, and the experiment was performed three times.

### 4.8. Ultrastructural Characteristics of the AgCl-NP Interaction with Pathogen

To observe the interaction of the AgCl-NPs with pathogenic bacterium cells, we observed morphological changes on the surfaces of bacterial cells by SEM after the AgCl-NPs treated bacterial cells with final concentration of 2.0 µg/mL^−1^ with shaking (200 rpm) at 30 °C for 4 and 8 h. Briefly, suspensions of the treated bacteria were centrifuged at 6000 rpm for 10 min at 4 °C, washed with PBS at pH 7, post-fixated in 1% (*w*/*v*) osmium tetroxide, and dehydrated in a graded ethanol series (50%, 70%, 80%, 90%, 95%, and 100%), as described by [53]. The samples were attached to an aluminum stub and sputter-coated with gold, and then examined and photographed in an SEM (Zeiss Gemini SEM 300, Germany). Suspensions of the bacteria treated by the CFCS extract with final concentration of the 2.0 µg/mL^−1^ were used as another control.

To observe the effect of the AgCl-NPs on the inner of pathogenic bacterial cells, we observed ultrastructural characteristics by using a JEM-1200EX transmission electron microscope (JEM-1200EX, Tokyo, Japan). Suspensions of the bacteria treated by the AgCl-NPs as described above and bacterial cells were fixed with glutaraldehyde (2.5%, *v*/*v*) in 0.1 M sodium phosphate buffer (pH 7.0) for 18 h at 4 °C. After centrifugation at 5000× *g* for 10 min, bacterial cells were embedded in potato dextrose agar (PDA). Afterward, samples were washed three times with phosphate-buffered saline (PBS) and fixed with (1% *w*/*v*, osmium tetroxide) for one hour at room temperature. The specimens were dehydrated for 15 min in a series of gradients ethanol concentrations (30 to 100%). After dehydrating in a graded ethanol series, samples were embedded in Spurr’s epoxy resin, as described by [57]. After the samples were cut with a Reichart-Jung Ultracute E ultramicrotome with a diamond knife, ultrathin sections collected on formvar-coated one-slot copper grids were stained with 2% uranyl acetate for 10 min and lead citrate for 5 min.

### 4.9. Statistical Analysis

All data reported in this study are the means of three replicates (*n* = 3). The analysis of the data was carried out using Statistic (version 8.1) software, and the means were compared by least significant difference (Fisher’s LSD) at the probability level (*p* ≤ 0.05) according to Steel and Torrie [58]. 

## 5. Conclusions

In the present study, we reported the biological, eco-friendly, and non-toxic method for the synthesis of AgCl-NPs using cell-free culture supernatant of bacterial strain IMA13. The biosynthesized AgCl-NPs were characterized by XRD analysis. The spherical and oval nanoparticles with 5–35 nm sizes were obtained. The strain IMA13 was identified as *B. mojavensis* on the basis of phylogenetic analysis using a combination of six gene sequences. MALDI-TOF analysis showed the lipopeptides compounds produced by strain IAM13 were attributed to the bacillomycin D, iturins, and fengycins. The antibacterial activity of the AgCl-NPs displayed the significant inhibition activity against growth, swarming, and swimming motility of phytopathogenic bacterium *R. solanacearum*, showing their potential application against bacterial wilt. The mode of AgCl-NPs action was characterized by SEM and TEM examinations according to AgCl-NP accumulation and hole formation on the surface of bacterial cells, as well as damage of cell walls and cytoplasmic membrane against bacterial cells, and on the basis of the morphological changes of bacterial cells inside and outside and the presence of nanoparticles confirmed by EDS, providing evidence of direct physical interaction between nanoparticles and bacterial cells. Overall, our findings suggest that biogenic AgCl-NPs could be considered as promising nanopesticides for disease management in eggplants. However, future investigations are needed to assess the antimicrobial potential of biogenic AgCl-NPs in realistic agricultural conditions such as field trials.

## Figures and Tables

**Table 1:** Primers used for PCR amplification.

Genes	Primers	Primer Sequence (5′–3′)
*16S*	27f	AGAGTTTGATCMTGGCTCAG
	1492r	GGYTACCTTGTTACGACTT
*gyrA*	42f	CAGTCAGGAAATGCGTACGTCCTT
	1066r	CAAGGTAATGCTCCAGGCATTGCT
*rpoB*	2292f	GACGTGGGATGGCTACAACT
	3354r	ATTGTCGCCTTTAACGATGG
*purH*	70f	ACAGAGCTTGGCGTTGAAGT
	1013r	GCTTCTTGGCTGAATGAAGG
*polC*	1505f	TTGTCGCTCAYAATGCAAGC
	2337r	YTCAAGCATTTCRTCTGTCG
*groEL*	550f	GAGCTTGAAGTKGTTGAAGG
	1497r	TGAGCGTGTWACTTTTGTWG

**Table 2:** The Bacillus strains used for the phylogenetic analysis.

Species of Bacillus	Strain	GenBank No.					
		*16S*	*gyrA*	*rpoB*	*purH*	*polC*	*groEL*
*Bacillus* sp.	IMA13	MZ310441.1	MZ338583.1	MZ338584.1	MZ338585.1	MZ338586.1	MZ338587.1
*B. sonorensis*	B-23154	NR_116189.1	EU138611.1	EU138818.1	EU138749.1	EU138680.1	EU138542.1
*B. pumilus*	NRS-272	NR_116191.1	EU138655.1	EU138862.1	EU138793.1	EU138724.1	EU138586.1
*B. atrophaeus*	NRS-213	NR_116190.1	EU138654.1	EU138861.1	EU138792.1	EU138723.1	EU138585.1
*B. subtilis*	NRS-744	NR_116192.1	EU138658.1	EU138865.1	EU138796.1	EU138727.1	EU138589.1
*B. subtilis* subsp. *spizizenii*	B-23049	NR_116187.1	EU138602.1	EU138809.1	EU138740.1	EU138671.1	EU138533.1
*B. subtilis* subsp.*inaquosorum*	B-23052	NR_116188.1	EU138605.1	EU138812.1	EU138743.1	EU138674.1	EU138536.1
*B. vallismortis*	B-14890	NR_116186.1	EU138601.1	EU138808.1	EU138739.1	EU138670.1	EU138532.1
*B. mojavensis*	B-14698	NR_116185.1	EU138598.1	EU138805.1	EU138736.1	EU138667.1	EU138529.1
*B. subtilis* subsp. *subtilis*	168	NC_000964.3:30279-31832	NC_000964.3: 6994-9459	NC_000964.3: 121919-125500	NC_000964.3: 708594-710132	NC_000964.3: 1727133-1731446	NC_000964.3: 650234-651868
*B. cereus*	ATCC 14579	NC_004722.1: 9187-10741	NC_004722.1: 6475-8946	NC_004722.1: 113908-117441	NC_004722.1: 309016-310551	NC_004722.1: 3794523-3790222	NC_004722.1: 257826-259460
*B. amyloliquefaciens*	ATCC 23350	NC_014551.1 9765-11318	NC_014551.1: 7010-9469	NC_014551.1: 122979-126560	NC_014551.1: 668235-669773	NC_014551.1: 1728311-1732624	NC_014551.1: 572972-574606
*B. licheniformis*	ATCC 14580	NC_006322.1: 9713-11250	NC_006322.1: 6900-9368	NC_006270.3: 120951-124532	NC_006322.1: 707082-708620	NC_006322.1: 1832492-1836808	NC_006322.1: 626726-628360
*B. amyloliquefaciens* subsp. *plantarum*	FZB42	NC_009725.1: 9760-11314	NC_009725.1: 7081-9462	NC_009725.1: 122626-126216	NC_009725.1: 670548-672086	NC_009725.1: 1642186-1646499	NC_009725.1: 619805-621439
*B. amyloliquefaciens*	Mito-5-13	AB610829.1	AB612173.1	AB615267.1	AB615405.1	AB612200.1	AB611006.1

## Supplementary Materials

The following supporting information can be downloaded online. Table S1: Lipopeptide compounds detected using MALDI-TOF mass spectrometry from bacterial strain IAM13.
