# Supplementary material for: Biosynthesis of Silver Chloride Nanoparticles by Rhizospheric Bacteria and Their Antibacterial Activity against Phytopathogenic Bacterium Ralstonia solanacearum
Source: Molecules. 2021 Dec 30;27(1):224. doi: 10.3390/molecules27010224 (PMC8746595; doi:10.3390/molecules27010224)
Supplement: Supplementary file 1 [file molecules-27-00224-s001.zip › molecules-1521880-supplementary.pdf]

Table S1. Lipopeptide compounds detected using MALDI-TOF mass spectrometry from bacterial strain IAM13

Mass peak (m/z)	Lipopeptides	precursor ions [M + X]	Refer.
1053.547	C14 bacillomycin D	[M+Na]+	[21]
1067.553	C15 bacillomycin D	[M+Na]+	[21]
1081.580	C16 bacillomycin D	[M+Na]+	[21]
1095.606	C17 bacillomycin D	[M+Na]+	[21]
1065.593	C14 iturin	[M+Na]+	[22]
1079.616	C15 iturin	[M+Na]+	[23]
1095.606	C15 iturin	[M+K]+	[22]
1109.619	C16 iturin	[M+K]+	[23]
1123.628	C17 iturin	[M+K]+	[22]
1152.727	C20 iturin	[M+Na]+	[22]
1435.875	C14 fengycin	[M+H]+	[24]
1449.901	C15 fengycin	[M+H]+	[24]
1463.922	C16 fengycin	[M+H]+	[24]
1473.844	C14 fengycin	[M+K]+	[25]
1477.932	C17 fengycin	[M+H]+	[24]
1485.901	C16 fengycin	[M+Na]+	[24]
1487.865	C15 fengycin	[M+K]+	[25]
1501.886	C16 fengycin	[M+K]+	[24]
1515.903	C17 fengycin	[M+K]+	[24]
1529.918	C16 fengycin	[M+K]+	[24]
